# Multi-View Deep Learning-Based Comprehensive Evaluation Model for Coating Protective Performance

**DOI:** 10.3390/ma19183969

**Published:** 2026-09-18

**Authors:** Yuming Tang, Suhang Hu, Dongliang Wu, Ziqiang Li, Wei Hu, Xuhui Zhao, Yu Zuo

**Affiliations:** 1 Key Laboratory of Carbon Fiber and Functional Polymer, Ministry of Education, Beijing University of Chemical Technology, Beijing 100029, China; tangym@mail.buct.edu.cn (Y.T.); 2024200522@buct.edu.cn (D.W.); l18797812485@163.com (Z.L.); zuoy@mail.buct.edu.cn (Y.Z.); 2 School of Information Science and Technology, Beijing University of Chemical Technology, Beijing 100029, China; husuhang26@stu.pku.edu.cn (S.H.); huwei@mail.buct.edu.cn (W.H.); 3 Institute of Medical Technology, Peking University Health Science Center, Peking University, Beijing, 100191, China

**Keywords:** organic coatings, protective performance, electrochemical impedance, deep learning, comprehensive performance evaluation

## Abstract

Organic protective coatings are extensively employed to mitigate metal corrosion, yet accurate quantitative evaluation of their performance degradation during service still poses a considerable challenge. This study aims to develop a multi-view deep learning framework for five-grade quantitative assessment of organic coating protective performance using routine electrochemical and mechanical parameters. This study aims to develop a multi-view deep learning framework for five-grade quantitative assessment of organic coating protective performance using routine electrochemical and mechanical parameters. Based on abundant laboratory-accelerated corrosion test data, independent single-view sub-models were constructed for three complementary descriptors, including the mid-frequency phase angle (*θ*_10 Hz_), open-circuit potential (OCP), and adhesion strength (*A*_s_). Prediction outputs from individual sub-models were fused through correlation-weighted voting, where weighting factors were determined by quantitative parameter-degradation correlations across various coating systems. The proposed framework achieves reliable five-level grading (excellent, good, fair, poor, failure) of coating protective performance. This methodology provides an effective data-driven framework for the predictive assessment of organic coating protective performance in practical engineering applications.

## 1. Introduction

Organic protective coatings are widely applied to metallic structures, including ships, offshore platforms, and large-scale bridges, to resist corrosion in harsh service environments, especially marine atmospheric and seawater conditions. Nevertheless, long-term exposure to water, oxygen, ultraviolet radiation, temperature cycling, and atmospheric pollutants continuously accelerates the degradation of organic coatings. Premature coating failure further induces substrate corrosion and substantially reduces the service life of metallic structural components. The degradation of organic coatings is dominated by medium penetration-induced failure mechanisms. In corrosive environments, reactive media such as water, oxygen, and corrosive ions gradually penetrate coating micro-pores and inherent defects, causing coating swelling, plasticization, and progressive deterioration of barrier performance. Continuous accumulation of corrosive substances at the coating–substrate interface triggers interfacial adhesion degradation, delamination, and blistering. Once the coating barrier fails, electrochemical corrosion initiates on the metal substrate, eventually leading to coating peeling and structural failure. Such degradation behavior is particularly prominent in marine environments, where high-concentration chloride ions significantly accelerate the overall failure process. Organic coatings exhibit diverse types and complex structural compositions, and their performance evolution is comprehensively governed by intrinsic material properties and external environmental factors. Although accurate evaluation of coating service status is essential for structural safety guarantee and scientific maintenance scheduling, quantitative and precise assessment of coating protective performance remains a challenging task.

Numerous studies have investigated the degradation behaviors and internal failure mechanisms of organic coatings and developed advanced performance evaluation and service life prediction methods. Natural outdoor weathering tests and laboratory-accelerated aging experiments are commonly combined with multiple characterization techniques, including electrochemical measurements (electrochemical impedance spectroscopy (EIS) and open-circuit potential (OCP)), physicochemical tests (gloss retention and adhesion strength measurement), and microscopic analysis (SEM and FTIR), to reveal dynamic degradation processes and establish standardized coating evaluation systems. Among these techniques, EIS is widely recognized as a dominant non-destructive testing method due to its convenient operation and low cost. Impedance spectra are fitted and analyzed to extract characteristic parameters that reflect coating deterioration degrees and substrate corrosion status, which effectively clarify coating failure mechanisms and quantify protective performance.

However, most existing studies are limited to controlled laboratory conditions, while field coating evaluation still relies heavily on subjective visual inspection, including discoloration, chalking, blistering, cracking, and rusting. Such qualitative evaluation criteria are highly empirical and incapable of accurate quantitative characterization. To address this issue, multiple quantitative evaluation indicators have been proposed in previous studies, such as low-frequency impedance modulus (|*Z*|_0.01 Hz_) [1,2], breakpoint frequency (*f*_b_) [3], single-frequency capacitance [4], and mid-frequency phase angle (*θ*_10 Hz_) [5,6] together with OCP [7,8]. In particular, the *θ*_10 Hz_ evaluation method proposed by Zuo et al. [5,6] exhibits high feasibility for field application. The protective level of organic coatings can be divided into five grades (excellent, very good, good, poor, and failure) based on the empirical correlation between *θ*_10 Hz_ and |*Z*|_0.01 Hz_ [9]. Nevertheless, single-parameter evaluation methods show limited universality for different coating systems, requiring further verification and optimization to improve evaluation reliability. Accordingly, multi-parameter comprehensive evaluation strategies have attracted increasing research attention. The integration of multi-dimensional characteristic indicators enables holistic characterization of coating degradation states and failure behaviors, effectively compensating for evaluation errors inherent in single-parameter methods.

A series of multi-parameter evaluation systems have been established in recent studies. Zhang et al. [10] constructed a correlation analysis model by synchronously processing electrochemical impedance, open-circuit potential, and Kelvin probe potential, and screened the optimal parameter combination for distinguishing coating degradation levels. Lee et al. [11] trained an artificial neural network based on theoretical impedance spectra of steel–coating composite systems and realized coating quality classification using breakpoint frequency (*f*_b_) and pore resistance (*R*_po_) parameters. Xu et al. [12] evaluated three typical coating states (intact, intermediate degradation, and complete failure) by extracting low-frequency impedance modulus (|*Z*|_0.01 Hz_), mid-frequency impedance modulus (lg *Z*_117 Hz_), mid-frequency phase angle (*θ*_10 Hz_) and break-point frequency (*f*_b_) from impedance data. Zhou et al. [13] established a coating corrosion evaluation system based on rough set theory, assigning weight coefficients to multiple visual and physical indicators, including gloss loss, color change, chalking, cracking, blistering, and peeling, to achieve quantitative classification of coating damage severity.

Furthermore, emerging technologies including digital image processing [14,15], electronic speckle pattern interferometry (ESPI) [16], terahertz (THz) detection [17,18] and artificial intelligence (AI) [19,20] have provided novel technical approaches for coating degradation monitoring and performance quantification. Advanced testing methods enable high-resolution detection of surface and internal micro-defects in coatings, while intelligent algorithms support automatic mining and analysis of massive experimental datasets. These techniques effectively identify complex nonlinear correlations between coating performance indicators and degradation kinetics, providing objective and reliable technical support for in-service coating performance evaluation [21].

Recent advances in materials informatics have demonstrated the great potential of well-structured deep-learning pipelines for material-related regression and classification tasks [22,23]. These representative works establish elegant standard practices for organizing multi-module workflows and presenting model architecture with high clarity, offering valuable references for data-driven material-performance modeling. However, such structured modeling paradigms have not yet been widely adapted to the quantitative performance evaluation of degradable organic protective coatings under corrosive service conditions.

Despite the significant progress in coating performance evaluation, several critical limitations remain in current research. First, most multi-parameter evaluation models adopt artificially selected characteristic parameters and empirically determined weight coefficients, which greatly restrict model adaptability across diverse coating systems and service environments. Second, the fusion analysis of multi-source heterogeneous data, including electrochemical, physicochemical, and visual indicators, is insufficient. The internal correlations between different parameters and their quantitative contributions to coating degradation remain unclear. Third, although AI-based intelligent evaluation methods show broad application prospects, most existing studies rely on single-modal experimental data or shallow neural network architectures, failing to fully exploit complementary effective information contained in multi-view test data. Therefore, a universal and systematic evaluation framework that can automatically extract hierarchical features from multi-source experimental data, quantify parameter importance, and adapt to various coating–environment systems is still lacking.

To address the above research gaps, multiple typical organic coating systems were tested under various laboratory-accelerated corrosion conditions in this work. A multi-view deep learning framework was developed to establish quantitative correlations between multi-dimensional experimental parameters and coating failure evolution, achieving comprehensive evaluation of coating protective performance. This study provides an efficient data-driven technical approach for predictive evaluation and health monitoring of organic coatings.

The main contributions of this work are summarized as follows:

- A five-level quantitative evaluation framework for coating protective performance is proposed, adopting low-frequency impedance |*Z*|_0.01 Hz_ as the core benchmark, combined with auxiliary indicators including mid-frequency phase angle, open-circuit potential, and adhesion strength.
- A multi-view deep learning evaluation architecture is constructed, in which independent single-view sub-models are established for individual indicators, and correlation-weighted voting fusion is implemented to realize automatic multi-parameter joint grading of coating degradation states.
- The proposed method is validated across multiple coating–substrate combinations under laboratory-accelerated corrosion scenarios, confirming its reliable prediction accuracy and cross-system generalization capability within the tested dataset range.

## 2. Materials and Methods

### 2.1. Materials and Sample Preparation

Q235 carbon steel and 5083 aluminum alloy panels with dimensions of 150 mm × 75 mm × 2.5 mm were utilized as metallic substrates in this work. Q235 carbon steel is a typical structural steel, with the main chemical composition (wt.%): C ≤ 0.22%, Mn ≤ 1.40%, Si ≤ 0.35%, S ≤ 0.050%, P ≤ 0.045%, and Fe in balance. It was commonly used for marine and offshore steel structural components. 5083 aluminum alloy is a typical Al–Mg alloy, with the main chemical composition (wt.%): Mg 4.0–4.9%, Mn 0.4–1.0%, Si ≤ 0.40%, Fe ≤ 0.40%, Zn ≤ 0.25%, Cu ≤ 0.10%, and Al in balance. It was widely applied for ship and offshore lightweight structures. All coating systems investigated in this study were anticorrosive organic coatings.

Surface pretreatment was performed according to ISO 8501-1:2007 standard [24]. Abrasive blasting yielded a Sa 2.5 near-white blast-cleaned surface, whereas intensive manual grinding produced a St3 thoroughly power-tool-cleaned surface. Coatings were deposited by airless spraying or brush-coating techniques. The dry-film thickness (DFT) of cured coatings was monitored using a DH-002 thickness gauge (Shenzhen DONGMEI Measuring Instrument Co., Ltd., Shenzhen, China). The target film thickness was strictly controlled at 80 ± 10 μm for all prepared coatings.

All coated specimens were cured for 7 days under ambient environmental conditions prior to accelerated corrosion testing. No fewer than seven parallel samples were prepared for each test condition to guarantee experimental reproducibility. A comprehensive dataset covering diverse coating formulations, substrate materials, and corrosion service environments was constructed for model training and validation. To simplify presentation, 10 representative single-layer anticorrosive coating systems with distinct compositions and service conditions were selected for systematic analysis and detailed elaboration. The test duration for these coating systems ranged from less than 100 days to more than 400 days. Partial typical test results are presented in subsequent sections, and detailed experimental parameters for all selected coating systems are summarized in Table 1.

### 2.2. Experimental Conditions

Three types of laboratory-accelerated corrosion tests were adopted to simulate marine service environments: constant-temperature immersion, neutral salt-spray test, and cyclic temperature-varying immersion. (i) Constant immersion test: Coated panels were continuously immersed in 3.5 % NaCl solution at room temperature (23 ± 2 °C). (ii) Neutral salt spray (NSS) test: Tests were conducted strictly following ASTM B117 standard. A 5% NaCl spray solution was used, the test temperature was maintained at 35 ± 2 °C, and solution pH was kept within 6.5–7.2 under continuous spraying. (iii) Cyclic temperature-varying immersion test: A periodic temperature alternation mode (40 °C for 12 h and 25 °C for 12 h) was employed to simulate diurnal temperature fluctuations of marine environments in southern China. The cyclic temperature procedure ran continuously throughout the test period. To stabilize the solution environment, the NaCl test solution was refreshed every 48 h to maintain constant chloride-ion concentration and pH value.

### 2.3. Measurements and Characterization Methods

#### 2.3.1. Morphological Observation

Surface macroscopic morphologies of coated specimens were periodically photographed and recorded during the whole corrosion exposure process. Degradation degrees of organic coatings were evaluated according to blistering and rusting grading criteria specified in national standard GB/T 1766–2008 [25].

#### 2.3.2. Electrochemical Impedance Spectroscopy (EIS) Test

Periodic EIS measurements were carried out using a PARSTAT 2273 electrochemical workstation (Princeton Applied Research, Princeton, NJ, USA). A standard three-electrode configuration was adopted: a saturated calomel electrode (SCE) served as the reference electrode, a platinum foil acted as the counter electrode, and the coated specimen functioned as the working electrode with an effective exposed area of 20 cm^2^. Before measurement, specimens were immersed in 3.5 % NaCl solution for 30 min to stabilize the open-circuit potential. EIS tests were performed at room temperature with an AC sinusoidal perturbation amplitude of 10 mV. The test frequency ranged from 10^−2^ to 10^5^ Hz, with 30 sampling points per frequency decade. The acquired impedance data were quantitatively fitted and analyzed using ZSimpWin software (V 3.50, Scribner Associates, Southern Pines, NC, USA).

#### 2.3.3. Open Circuit Potential (OCP) Test

The open-circuit potential (*E*_ocp_) of coated specimens was measured with a MAS830L digital multimeter (Shenzhen HUAYI Instrument Co., Ltd., Shenzhen, China). Measurements were performed in 3.5 % NaCl solution at room temperature. The coated specimen and SCE were used as the working electrode and reference electrode, respectively. Potential data were recorded after 30 min immersion to stabilize the electrochemical system and obtain steady-state OCP values.

#### 2.3.4. Adhesion Strength Testing

Interface adhesion strength between coatings and substrates was measured via pull-off tests in accordance with GB/T 5210-2006 [26]. A two-component structural adhesive (Ergo 1690, Kisling AG, Wetzikon, Switzerland) was mixed at a mass ratio of 1:10 (component A: component B) and degassed to remove internal bubbles. Aluminum test coupons 20 mm in diameter were bonded onto coating surfaces and cured for 24–36 h under ambient conditions. A PosiTest AT-M adhesion tester (DeFelsko Corporation, Ogdensburg, NY, USA) applied tensile load at a constant rate of 0.2 MPa/s until coating detachment. The peak tensile force recorded at failure was defined as coating adhesion strength (MPa). Six parallel tests were performed for each group of specimens, and final results were reported as mean value ± standard deviation.

## 3. Results and Discussion

### 3.1. Accelerated Corrosion Test Results

**Figure 1:**
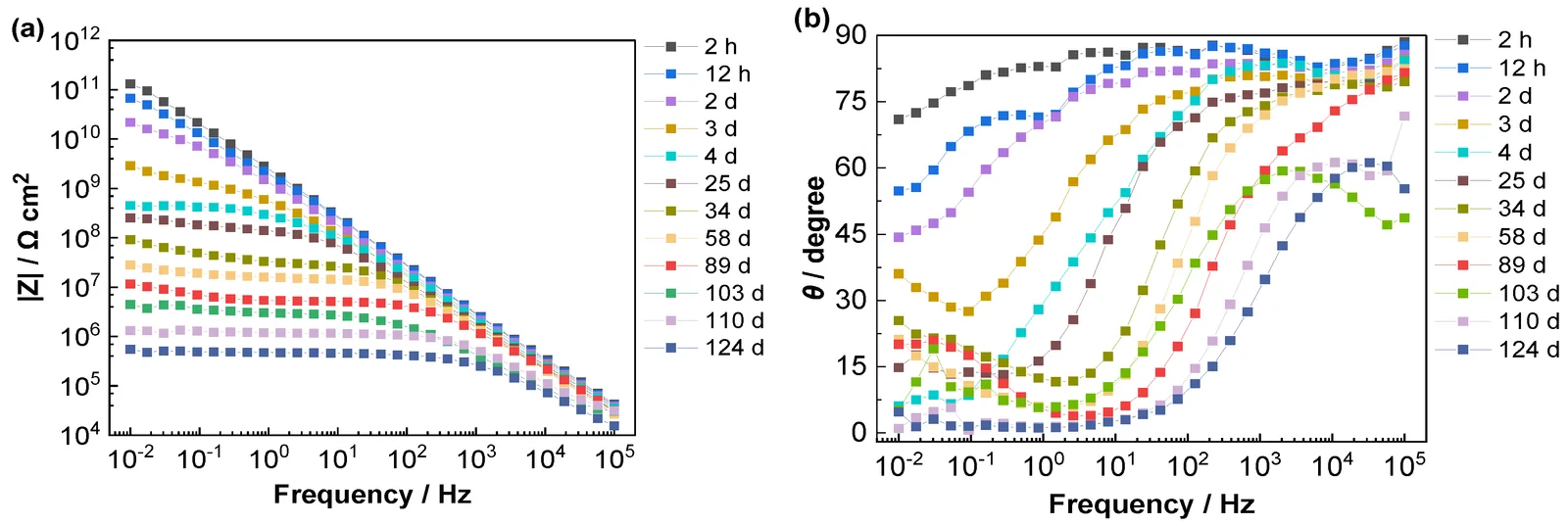
EIS spectra of Coating System No. 1 during continuous immersion in 3.5 wt% NaCl solution: (**a**) Nyquist plot; (**b**) Bode plot.

The corrosion degradation behavior and protective performance of organic coating systems were illustrated using Coating System No. 1 as a typical research object. The electrochemical impedance spectroscopy (EIS) spectra of solvent-free epoxy coatings on Q235 steel substrates during a 124 d. immersion period in 3.5 % NaCl solution are displayed in Figure 1. The continuous penetration of corrosive electrolyte alters the internal dielectric characteristics of coating films, which induces regular variations in coating resistance and capacitance. Coating resistance (*R*_c_), which is acquired by fitting EIS data with a reasonable equivalent circuit model, is widely regarded as a core indicator for evaluating the barrier performance of organic coatings. In comparison, low-frequency impedance modulus (|*Z*|_0.01 Hz_) can be directly extracted from original impedance spectra without relying on equivalent circuit assumptions, and thus is frequently adopted for rapid and quantitative coating performance evaluation.

**Figure 2:**
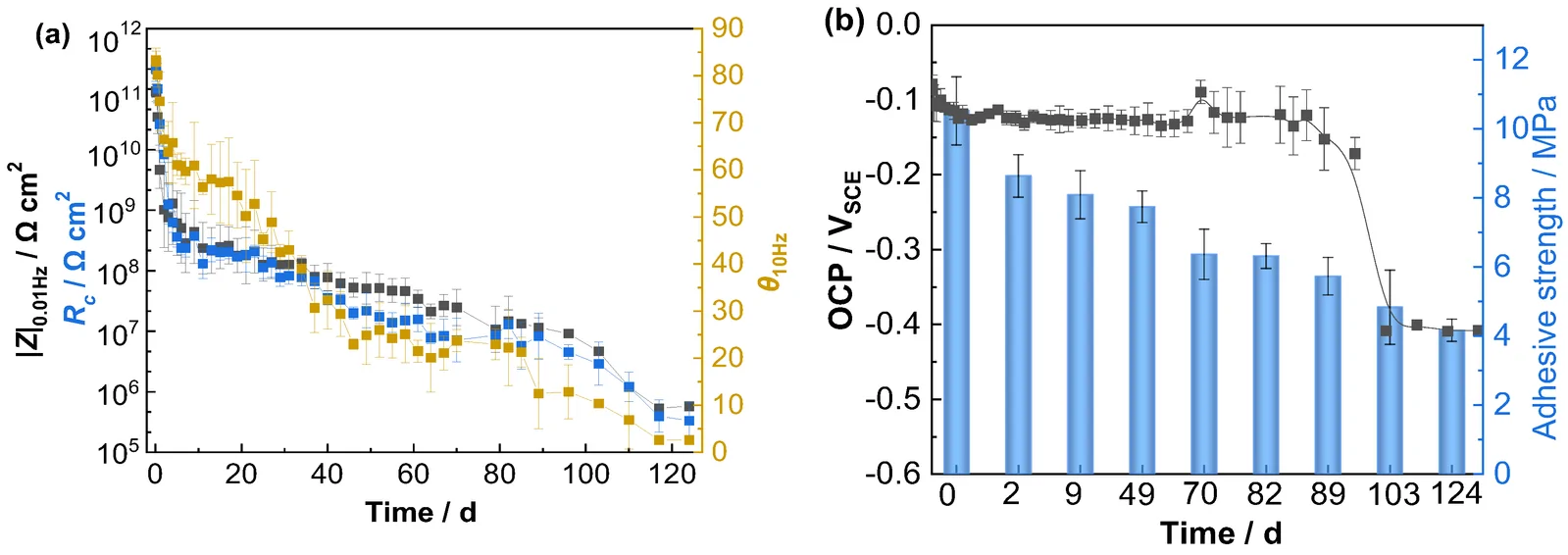
Comparative evolution of coating characteristic parameters for Coating System No.1: (**a**) |*Z*|_0.01 Hz_, *R*_c_ and *θ*_10 Hz_; (**b**) OCP and adhesion strength.

As depicted in Figure 2a, the temporal evolution trends of |*Z*|_0.01 Hz_ and *R*_c_ were highly consistent with comparable numerical magnitudes throughout the entire immersion process. This excellent consistency verified that |*Z*|_0.01 Hz_ could be used as a reliable substitute for *R*_c_ to characterize the barrier deterioration of organic coatings. Additionally, the mid-frequency phase angle (*θ*_10 Hz_) exhibited a similar declining trend to the above impedance parameters, which further confirmed its feasibility as a complementary indicator for evaluating coating degradation. Open-circuit potential (OCP) was adopted to reflect the integrity of coating barrier structures, where a more positive OCP value corresponds to a more complete insulating coating state with lower corrosion risk. Interface adhesion strength is another critical physical indicator for assessing the long-term protective performance of metal-coating systems. The dynamic evolution of OCP and adhesion strength during corrosion exposure is summarized in Figure 2b. The OCP values remained relatively stable in the early and middle immersion stages, but a sharp decline was observed in the late exposure period, which indicated that the corrosive electrolyte penetrated through the coating film and reached the coating–substrate interface. Meanwhile, the adhesion strength of the coating decreased continuously with the extension of corrosion time. This evolution behavior was consistent with the attenuation trend of impedance parameters (|*Z*|_0.01 Hz_ and *R*_c_), which was attributed to the gradual damage of interfacial bonding caused by osmotic blistering and cumulative corrosion product deposition.

**Figure 3:**
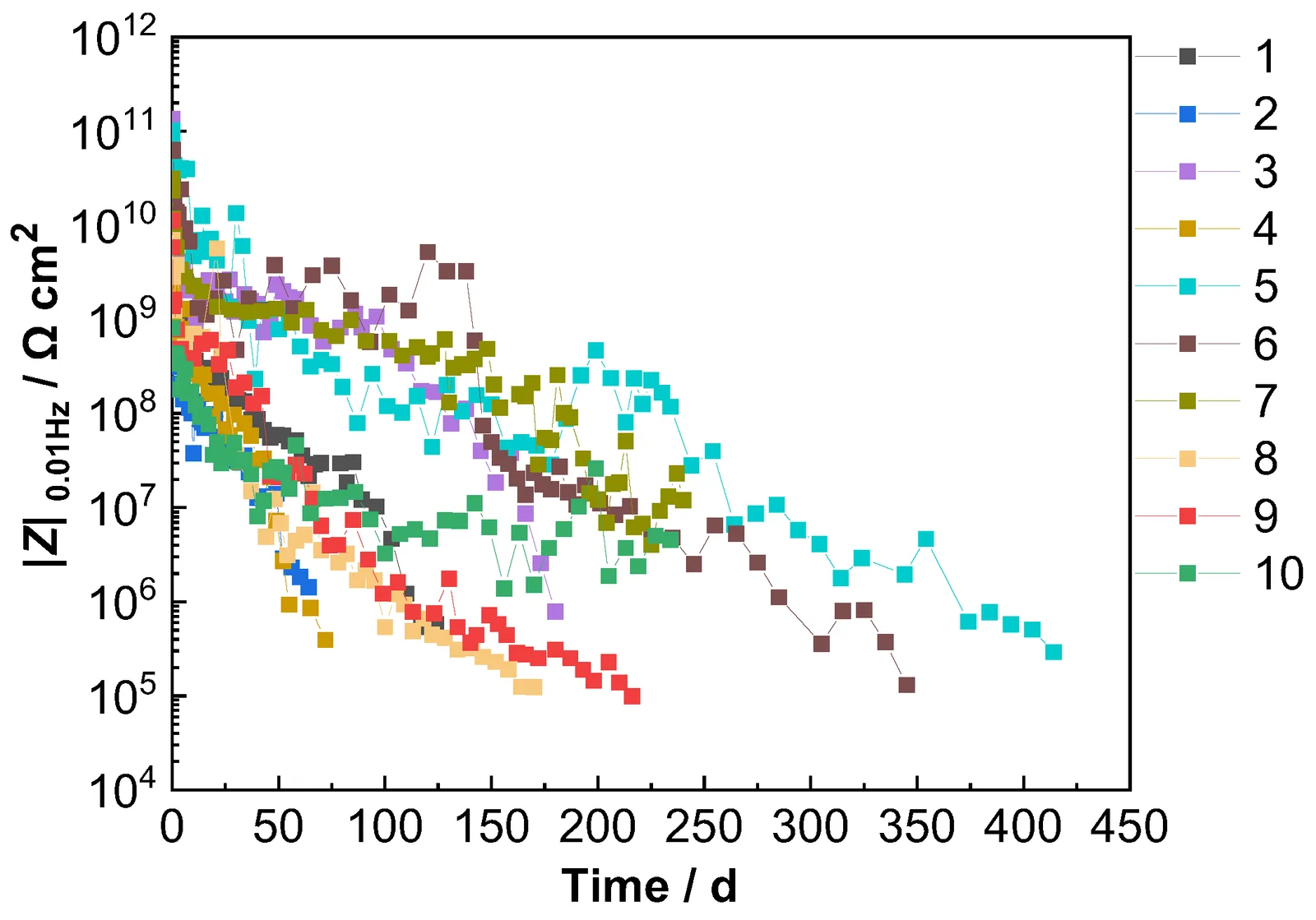
Temporal variations of |*Z*|_0.01 Hz_ for all coating samples during corrosion testing.

**Figure 4:**
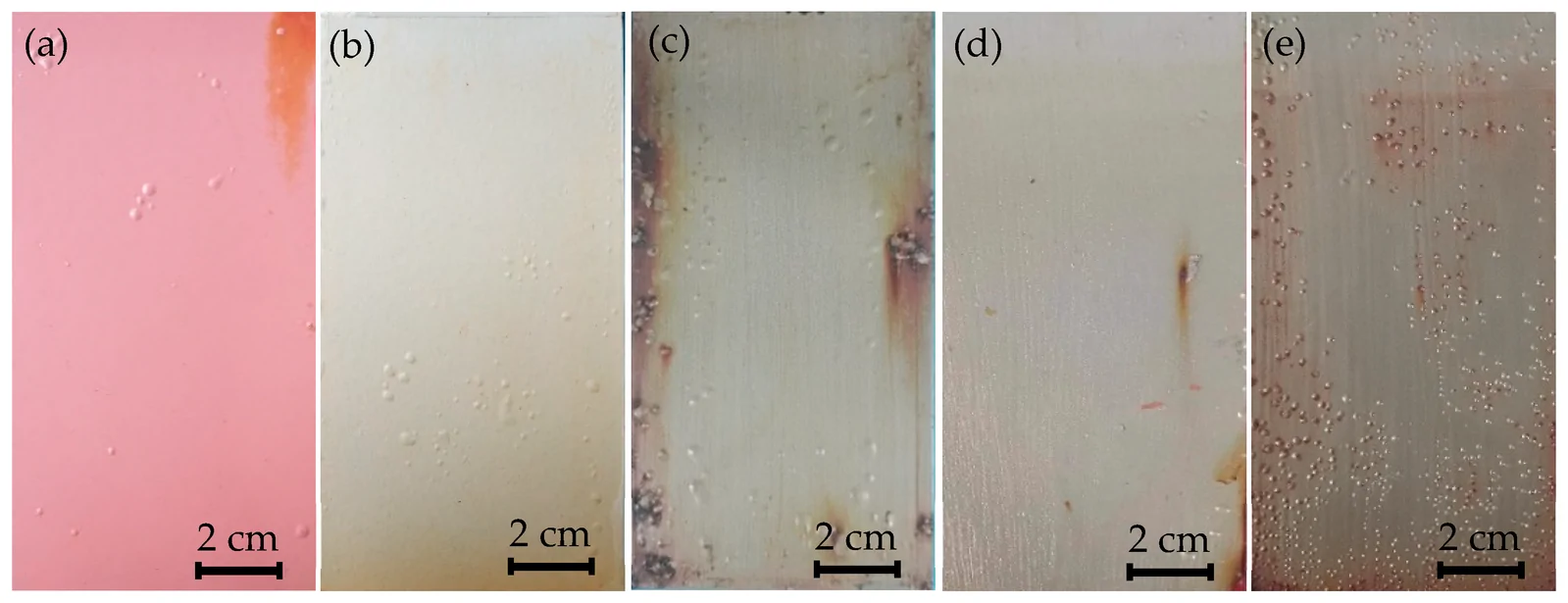
Macroscopic morphologies of typical coating samples after accelerated corrosion testing: (**a**) Sample 1 after 124 d of immersion; (**b**) Sample 4, after 72 d of immersion; (**c**) Sample 5, after 404 d of immersion; (**d**) Sample 7 after 225 d of immersion. (**e**) Sample 8 after 170 d of immersion.

Considering the manuscript length limitation and the consistency of experimental rules and analytical mechanisms with previous studies [27], the detailed time-dependent degradation results of each individual coating system were not presented in this section. Similarly, the full original datasets of all coating groups listed in Table 1 were omitted for brevity. The overall evolution of low-frequency impedance modulus (|*Z*|_0.01 Hz_) for all 10 coating systems during accelerated corrosion tests is summarized in Figure 3. At the end of the accelerated test period, the |*Z*|_0.01 Hz_ values for all coating samples dropped below 1.0 × 10^6^ Ω·cm^2^, which demonstrated the severe deterioration of coating barrier performance and substantial coating failure. The macroscopic surface morphologies of typical coating specimens after corrosion testing are exhibited in Figure 4. Blistering and substrate rusting were verified as the dominant failure modes for all investigated single-layer coating systems under marine corrosive environments. Accordingly, the subsequent sections focus on the elaboration of the multi-view deep learning framework and the detailed construction procedure of the coating protective performance comprehensive evaluation model.

### 3.2. Characterization Parameters During Coating Service Life

In-depth analysis of experimental data acquired from multiple metal-coating systems under accelerated corrosion conditions demonstrated that typical characteristic parameters exhibited regular evolutionary behaviors during coating degradation. The evaluated indicators covered electrochemical parameters (|*Z*|_0.01 Hz_, OCP, and *θ*_10 Hz_) and the mechanical property (adhesion strength), all of which could effectively reflect the continuous deterioration of coating protective performance and serve as reliable evaluation indicators for coating failure assessment [28]. The correlation strength of each parameter with coating protective performance varied significantly among different coating systems and service environments, and the corresponding weight coefficients of individual indicators could be quantified via mathematical correlation analysis (e.g., Pearson correlation analysis). Moreover, the selection of dominant evaluation parameters was substantially affected by corrosion environmental conditions. Specifically, electrochemical parameters played a decisive role in coating performance characterization under constant immersion and neutral salt-spray environments. In contrast, the correlation-based weighting factor of adhesion strength became significantly larger under cyclic temperature–humidity alternating conditions, which was mainly attributed to interfacial bonding deterioration induced by periodic thermal stress. Accordingly, both intrinsic coating characteristics and external service-environmental factors should be comprehensively considered to achieve accurate, stable, and reliable quantitative evaluation of coating protective performance.

## 4. Development of a Comprehensive Coating Performance Evaluation Model

A multi-parameter integrated evaluation framework coupled with qualitative grading criteria was developed to quantify the protective capacity of organic coatings via systematic statistical analysis of multiple characteristic parameters. Four sequential core procedures were implemented for model construction: (1) Selection of baseline characteristic indicator: The low-frequency impedance modulus (|*Z*|_0.01 Hz_) was screened as the baseline metric for qualitative grading of coating protective performance. The reliability of this indicator was validated by abundant accelerated corrosion experimental data, and tiered performance boundaries were defined according to its magnitude intervals. (2) Determination of grading threshold intervals: On the basis of the grading rules of the baseline indicator, synchronous time-series matching was adopted to calibrate the corresponding classification intervals for auxiliary characteristic parameters, including mid-frequency phase angle, open-circuit potential, and adhesion strength. (3) Calculation of parameter weight coefficients: An optimized Pearson correlation analysis method was adopted to quantify the linear correlation between |*Z*|_0.01 Hz_ and all other characteristic parameters. Weight coefficients were assigned to each parameter in accordance with the magnitude of correlation degrees to reflect their relative correlation strengths with coating degradation evaluation. (4) Construction of multi-view deep learning evaluation architecture: A deep neural network embedded with multi-view feature fusion and weighted voting mechanisms was established. Comprehensive multi-dimensional parameter inputs were imported into the network to realize automatic qualitative tiered judgment of coating protective performance.

### 4.1. Baseline Indicator

On the basis of previous theoretical investigations and corrosion experimental studies [2,5,29,30], the low-frequency impedance modulus (|*Z*|_0.01 Hz_) is widely recognized as a reliable quantitative indicator for characterizing the barrier performance of organic anticorrosive coatings. This metric was thereby selected as the baseline classification indicator for coating performance assessment in the present work. By combining previously reported literature and our own experimental work [5,9,30,31], a five-tier coating performance grading standard and the corresponding threshold values of|*Z*|_0.01 Hz_ were formulated, as listed in Table 2.

It is worth noting that, strictly speaking, coating failure criteria are governed by multiple factors including coating material, metallic substrate, exposure environment, and coating thickness. Therefore, an accurate low-frequency impedance threshold for a given coating-environment combination should be established based on substantial corrosion experiments and cross-validation among multiple performance measurements. Nevertheless, supported by the extensive published literature and our preliminary research findings, the value of 1 × 10^6^ Ω·cm^2^ can serve as an approximate criterion for coating failure or near-failure of ordinary organic protective coatings, such as epoxy coatings free of conductive pigments (e.g., zinc and magnesium powders), under common service environments such as marine conditions.

**Figure 5:**
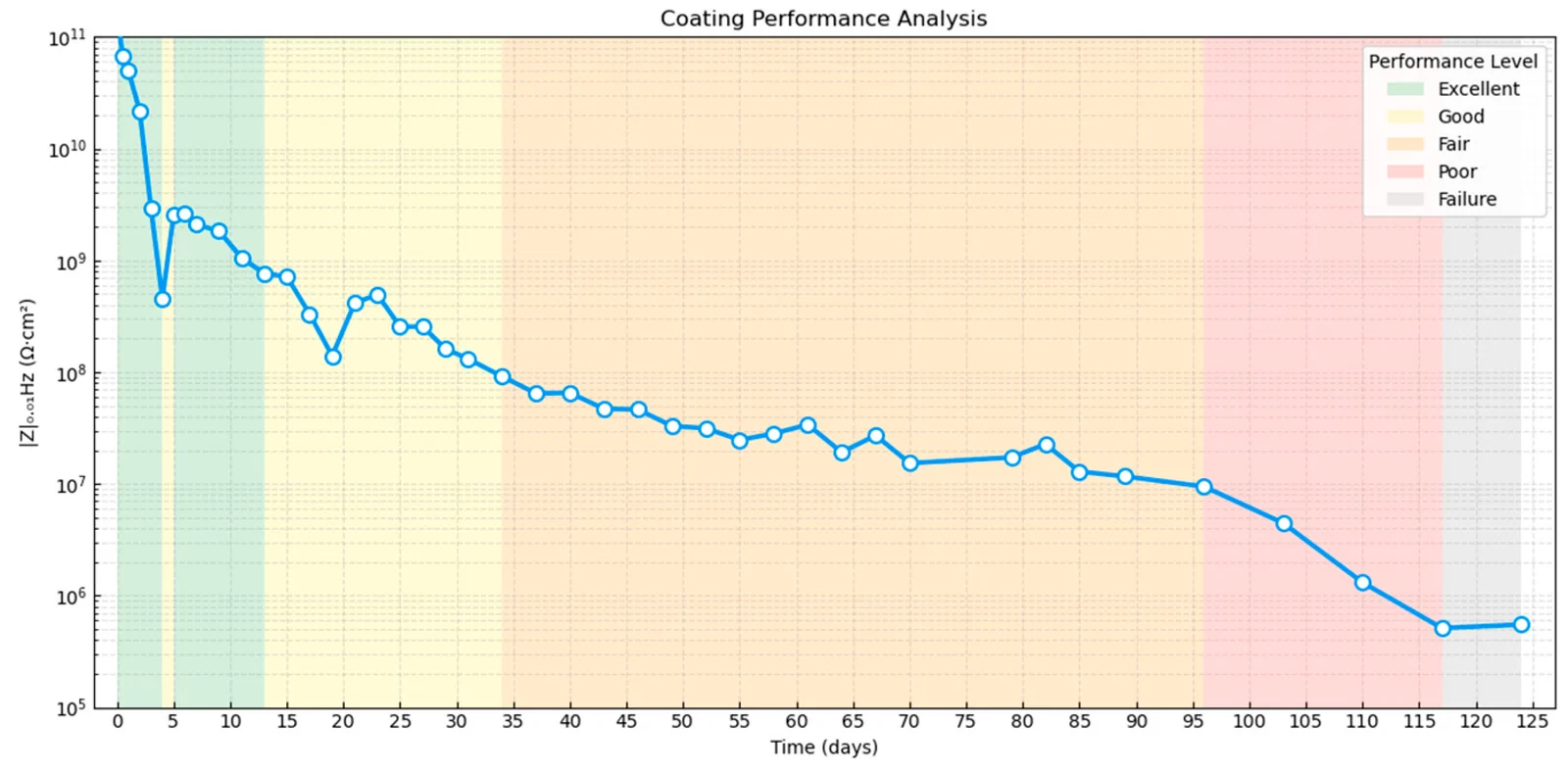
Temporal evolution of |*Z*|_0.01 Hz_ and performance grading during the degradation of Coating System No.1.

With this grading framework established, as a representative example for Coating System No. 1, the temporal evolution of **|*****Z*****|**_0.01 Hz_ over exposure time is illustrated in Figure 5. The impedance curve generally shows a monotonically decreasing trend, and the degradation of coating protective performance is interpreted according to the above-mentioned five-level criteria. Five distinct color labels are used in the figure to denote the five performance tiers: excellent, good, fair, poor, and failure.

### 4.2. Grading Threshold Intervals for Auxiliary Parameters

Taking |*Z*|_0.01 Hz_ as the baseline indicator, the unified five-tier performance grading criteria were mapped onto other characteristic parameters via time-axis synchronous alignment to obtain their respective classification thresholds. Coating System No. 1 was selected as a typical case. Time-series curves of |*Z*|_0.01 Hz_ and three auxiliary parameters (mid-frequency phase *θ*_10 Hz_, open-circuit potential OCP and adhesion strength *A*_s_) were aligned on a unified time axis. Quantitative correlations between each auxiliary parameter and coating performance tiers were extracted from synchronized degradation timelines. The calibrated threshold intervals for all characteristic parameters are summarized in Table 3. Threshold analysis from Table 3 indicated that although individual auxiliary parameters could partially reflect coating degradation, their non-monotonic evolution produced inconsistent linear correlations against the baseline |*Z*|_0.01 Hz_. This inconsistency manifested as overlapping numerical ranges belonging to different performance grades. Therefore, reliable and unambiguous identification of coating degradation states could not be achieved by relying on any single characteristic parameter alone. Such overlapping-threshold behavior was observed for all tested single-layer coating systems under various accelerated-corrosion environments.

### 4.3. Weighting Factor Analysis

Curve correlation analysis was performed to quantify the correlation-based weighting factor of each characteristic parameter with respect to coating protective performance. Taking |*Z*|_0.01 Hz_ as the baseline reference indicator, the weighting factor (*λ*_i_) for each parameter (*i*) was calculated from its correlation with |*Z*|_0.01 Hz_. This correlation-based calculation strategy evaluates how suitable each parameter is as a quantitative metric for coating-degradation assessment.

Several curve-correlation algorithms were compared in this study. A modified algorithm derived from the Pearson correlation coefficient was finally adopted to compute the correlation-based weighting factor (*λ*_i_). Geometrically, the Pearson correlation coefficient quantifies shape similarity between two time-series curves [32,33]. Nevertheless, experimental results showed that some characteristic parameters did not follow the monotonically decreasing temporal trend of the baseline |*Z*|_0.01 Hz_; instead, several indicators exhibited an increasing variation tendency, such as water uptake and porosity of coatings. Consequently, the original Pearson correlation coefficient cannot properly reflect the intrinsic correlation between each parameter and coating protective performance under such divergent trend patterns. An improved Pearson correlation approach was therefore proposed to address this limitation. For each characteristic parameter, Pearson coefficients were computed separately between the |*Z*|_0.01 Hz_ curve a and the original parameter curve, as well as its transformed counterpart. The maximum absolute value of these two coefficients was taken as the final correlation-based weighting factor (*λ*_i_). Weighting factors obtained by this method were normalized within [0, 1], where larger values indicate stronger influence on coating degradation.

(1) Reciprocal transformation for the parameter curves

For parameters whose trends opposed the monotonically decreasing baseline of |*Z*|_0.01 Hz_ (e.g., water uptake), reciprocal transformation via Equation (1) was applied to align their variation trends with the baseline. A small positive constant *ε* = 10^−8^ was introduced to prevent division-by-zero numerical singularities.

(1) $$X^{\prime }(t)=\frac{1}{X\left(t\right)+\varepsilon }$$

(2) Dual correlation coefficient calculation

Pearson correlation coefficients between the |*Z*|_0.01 Hz_ baseline and the original and reciprocal-transformed parameter curves were calculated using Equations (2) and (3), respectively.

(2) $$\begin{array}{cc} \rho_{i}^{\mathrm{original}} & =\frac{\sum_{t=1}^{T}\left(X_{i}(t)-\bar{X_{i}}\right)\left(Y(t)-\bar{Y}\right)}{\sqrt{\sum_{t=1}^{T}{\left(X_{i}(t)-\bar{X_{i}}\right)}^{2}}\cdot \sqrt{\sum_{t=1}^{T}{\left(Y(t)-\bar{Y}\right)}^{2}}} \end{array}$$

(3) $$\begin{array}{cc} \rho_{i}^{\mathrm{mirror}} & =\frac{\sum_{t=1}^{T}\left(X_{i}^{\mathrm{mirror}}(t)-\bar{X_{i}^{\mathrm{mirror}}}\right)\left(Y(t)-\bar{Y}\right)}{\sqrt{\sum_{t=1}^{T}{\left(X_{i}^{\mathrm{mirror}}(t)-\bar{X_{i}^{\mathrm{mirror}}}\right)}^{2}}\cdot \sqrt{\sum_{t=1}^{T}{\left(Y(t)-\bar{Y}\right)}^{2}}} \end{array}$$

(3) Weighting factor extraction

The preliminary weighting factor (*λ*_i_^′^) was defined in Equation (4), which selected the maximum absolute value from the two computed correlation coefficients.

(4) $$\lambda_{i}^{\prime }=\max\left(\rho_{i}^{\mathrm{original}},\;\rho_{i}^{\mathrm{mirror}}\right)$$

Based on this improved Pearson algorithm, correlation analysis between each core characteristic parameter and coating protective performance was carried out using |*Z*|_0.01 Hz_ as the benchmark. The resulting weighting-factor values for all coating systems are summarized in Table 4. Key conclusions are listed below:

① The average weighting factor (*λ*) of adhesion strength (*A*_s_) exceeded 0.8, indicating a strong correlation between adhesion strength and coating degradation. Adhesion strength was thus identified as a primary evaluation parameter.

② The weighting factors for the mid-frequency phase angle (*θ*_10 Hz_) and open-circuit potential (OCP) were mostly higher than 0.6. Although their correlation strengths were slightly lower than that of adhesion strength, they featured short testing durations and non-destructive detection, making them important indicators for on-site field evaluation of coating protective performance.

### 4.4. Construction of Multi-View Deep Learning Comprehensive Evaluation Model

A multi-view deep-learning integrated evaluation framework was developed to quantitatively grade coating protective performance by jointly analyzing multi-dimensional characteristic parameters. The overall model consisted of three modular components: (1) data preprocessing module, (2) multi-view neural-network training module, and (3) multi-view weighted-voting fusion module.

(1) Data preprocessing module

Time-series data of core characteristic parameters covering full coating-degradation lifecycles were assembled to construct the model-input dataset. Input indicators included the electrochemical mid-frequency phase angle (*θ*_10 Hz_), open-circuit potential (OCP), and mechanical adhesion strength (*A*_s_). These parameters jointly provided multi-perspective characterization of coating performance degradation.

Standardization and normalization were implemented to eliminate numerical discrepancies originating from heterogeneous units and value ranges among different parameters, thus improving training convergence and prediction accuracy. For parameters with wide-ranging values such as impedance-related indices, logarithmic transformation followed by Z-score standardization was applied. Other time-series variables were linearly scaled to the interval [0, 1] using Min–Max scaling. After uniform normalization, all characteristic parameters were converted into comparable numerical scales to stabilize model training and accelerate convergence.

(2) Multi-view neural network training module

**Figure 6:**
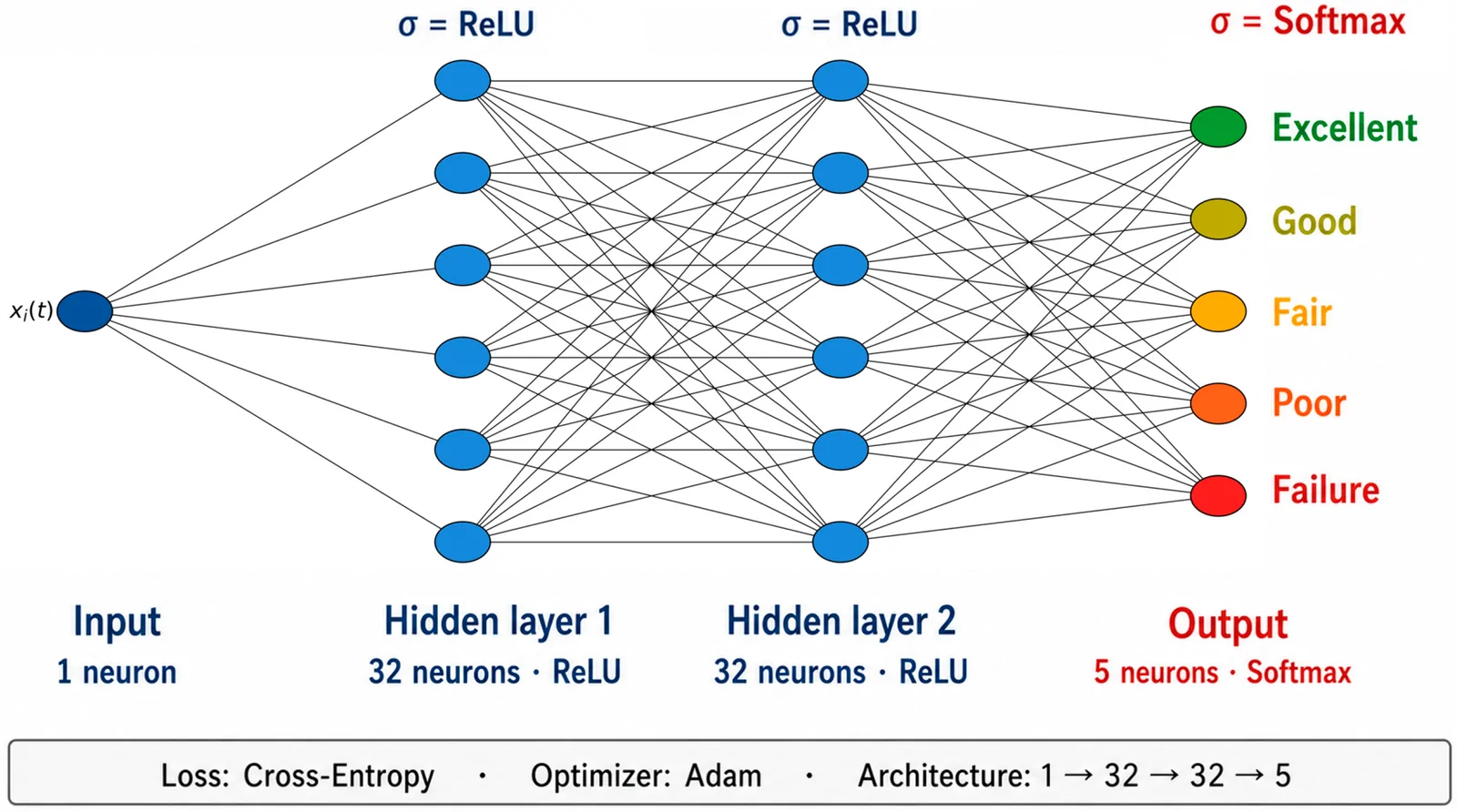
Schematic diagram of the four-layer multilayer perceptron (MLP) sub-model architecture adopted in this work.

Independent neural-network sub-models were built for each characteristic parameter to enable performance grading based on individual-indicator features. Each sub-model adopted a four-layer multilayer perceptron (MLP) with a 1-32-32-5 topological, as illustrated in Figure 6. The input layer contained one neuron, which received the measured value of a single characteristic parameter at one measurement instant. Each training sample corresponded to one synchronized parameter-time measurement point. Accordingly, each sub-model learned a point-wise mapping from parameter value to five-tier performance grade across the full degradation process, rather than extracting time-series features from individual samples. Two hidden layers with 32 neurons each were utilized to capture nonlinear degradation relationships from input data. The output layer contained five neurons corresponding to the five coating-performance grades: excellent, good, fair, poor, and failure. The Rectified Linear Unit (ReLU) activation function was employed for all hidden layers to enhance nonlinear fitting capability, whereas the Softmax activation function in the output layer produced probability distributions over performance grades. During training, cross-entropy loss and the Adam optimizer were adopted, both of which are standard deep-learning algorithms [34,35]. Backpropagation iteratively updated network weights until stable prediction of coating-degradation grades was attained.

(3) Multi-view weighted voting mechanism module

The multi-view weighted-voting strategy was developed based on ensemble-learning and multi-expert-model fusion theory. Task decomposition mitigated the curse of dimensionality and avoided negative-transfer effects induced by high-dimensional mixed inputs, improving the generalization and anti-noise robustness of the overall evaluation model.

Each parameter-specific MLP sub-model ran independently and output two pieces of information: a predicted coating-performance grade and its corresponding prediction probability. Pre-calculated weighting factors (*λ*), derived from the correlation between each parameter and the baseline |*Z*|_0.01 Hz_, serve as voting coefficients. Parameters with larger *λ* values, which reflected stronger correlation with coating protective performance, were endowed with higher voting weights. The comprehensive evaluation grade was determined via the following weighted-fusion procedure. For a given performance grade *g*, the comprehensive voting probability *P*(*g*) was calculated by normalized weighted summation:

(5) $$P(g)=\frac{\sum \lambda_{i}p_{i}(g)}{\sum \lambda_{i}}$$

where *λ*_i_ denotes the weighting factor of the *i*-th sub-model, and *p*_i_(*g*) represents the prediction probability of grade *g* output by the *i*-th sub-model.

For each predefined performance grade, the prediction probabilities output by individual sub-models were multiplied by their respective weighting factors. The weighted probabilities from all sub-models were summed to compute the comprehensive voting score for that grade. The final coating-performance grade corresponded to the grade with the maximum comprehensive voting score.

**Figure 7:**
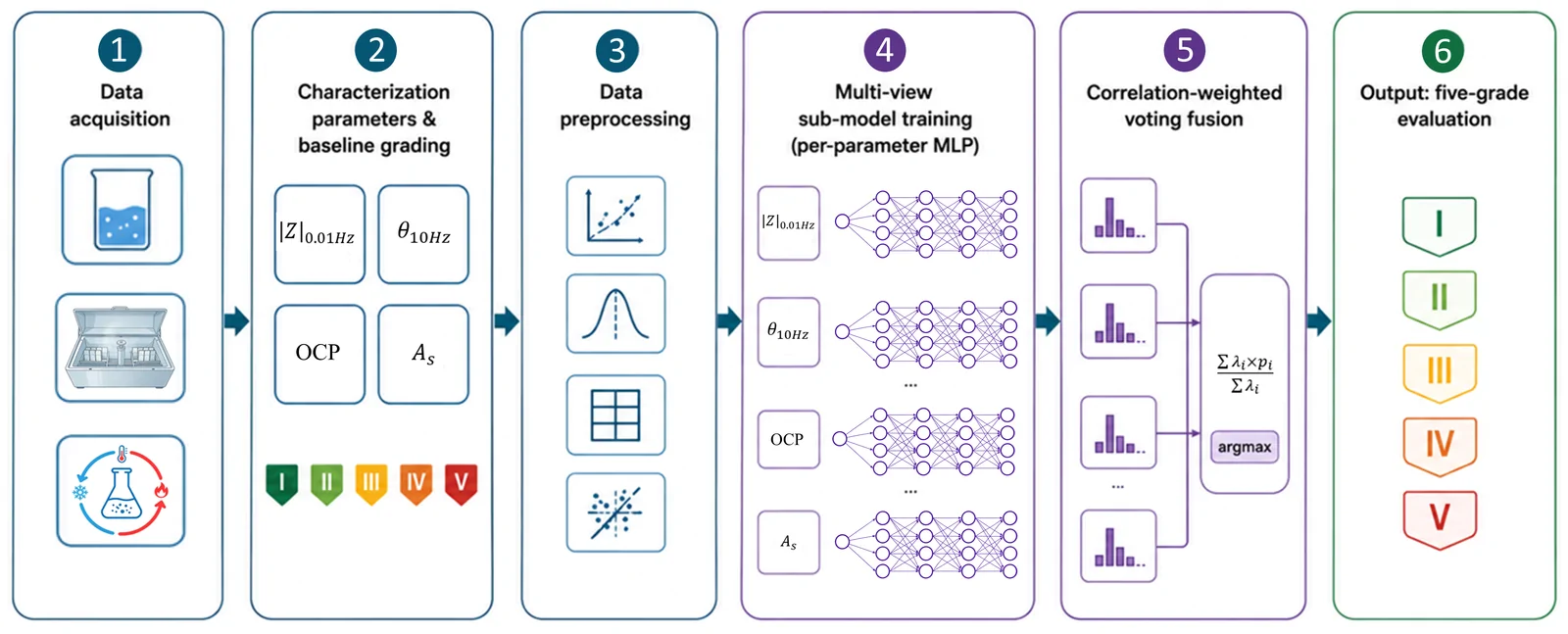
Overall workflow of the multi-view deep-learning-based model for comprehensive protective-performance evaluation of organic coatings, showing the full pipeline from experimental data input to five-grade prediction output.

It should be noted that correlation analysis for all impedance-derived parameters yielded high weighting factors for several indicators. Nevertheless, only one most-representative impedance parameter with optimal correlation is recommended for integration into the voting module. This impedance metric is fused with other physicochemical and mechanical parameters to build a stable, high-precision multi-view evaluation model. The complete end-to-end workflow of the multi-view deep-learning-based coating-evaluation approach, covering experimental data acquisition, parameter measurement, data preprocessing, per-parameter sub-model training, correlation-weighted voting fusion, and final five-tier performance prediction, is summarized schematically in Figure 7.

## 5. Model Validation

The multi-view deep-learning model for integrated quantitative evaluation of coating protective performance was validated from two dimensions: prediction accuracy and generalization performance. Prediction accuracy was verified by comparing model-output grades against actual measured corrosion test results. Generalization capability was assessed via cross-system and cross-environment tests covering diverse substrate alloys, coating formulations, neutral salt-spray and long-term immersion accelerated-corrosion environments. This validation workflow was designed to examine the generalization potential of the constructed model to different coating systems under laboratory-simulated marine corrosive conditions.

### 5.1. Dataset Partitioning and Validation Scheme

Considering metal-coating degradation mechanisms and time-dependent failure characteristics, a two-tier validation scheme was established to evaluate model robustness and generalization ability: (1) within-system stratified hold-out validation for sub-model training and selection; (2) cross-system and cross-environment verification across the ten coating systems. The detailed implementation procedures are described below:

(1) Acquisition of multi-source characteristic data: Full time-series monitoring data covering the whole degradation lifecycle were collected for each single-layer coating system under corresponding accelerated corrosion environments (Table 1). Multiple electrochemical and mechanical performance indicators were recorded under different corrosive exposure conditions.

(2) Within-system hold-out partitioning strategy: For each coating system, 80% of valid measurement points were randomly assigned to the system-specific training set, and the remaining 20% formed the hold-out set. The training data contained continuous full-lifecycle monitoring records consisting of discrete time-point parameter data. Standard preprocessing was performed to ensure data integrity and precision; no interpolation was applied to the data fed into the grading model. Outlier detection and removal were conducted using Grubbs’ test at a significance level of α = 0.01, and points exceeding the ±3σ threshold were discarded to mitigate measurement noise.

Hold-out points for each system were sampled at randomly scattered time nodes (e.g., 7 d, 80 d, 350 d) from synchronized time-series records. This breaks temporal correlation with training points and ensures the hold-out set covers the full coating evolution from the intact initial state to substrate corrosion failure. Furthermore, all measurements originating from the same specimen or experimental batch were assigned entirely to one partition, implementing specimen- and batch-level isolation; no measurements from training specimens or batches appeared in the hold-out set. The ten coating systems in Table 1 were prepared independently and covered different substrate materials, coating formulations and accelerated corrosion modes (constant-temperature immersion, neutral salt spray, cyclic temperature-variant immersion). Within-system training-hold-out was executed for every system, and per-system results were cross-examined to realize cross-system and cross-environment verification. This evaluation strategy achieved a strictness comparable to leave-one-coating-system-out validation. T.

### 5.2. Validation Results and Analysis

Validation and quantitative analysis of the multi-view deep-learning model were carried out strictly following the stratified dataset-splitting strategy described above. Coating System No. 1 was selected as a typical case. After model training on its corresponding training dataset, weight coefficients for major characteristic parameters are listed in the first row of Table 4. The analytical results showed that adhesion strength (*A*_s_) yielded a high correlation-based weighting factor (*λ* = 0.93). The mid-frequency phase angle (*θ*_10 Hz_) produced a considerable weighting factor (*λ* ≈ 0.6), while the weighting factor for OCP was 0.37. Three indicators (*A*_s_, *θ*_10 Hz_ and OCP) or two indicators (*A*_s_ and *θ*_10 Hz_) were adopted for model verification at five representative immersion instants (2 d, 9 d, 27 d, 89 d, 124 d). As shown in Table 5, for both indicator combinations, model-predicted performance grades agreed with benchmark grades derived from |*Z*|_0.01 Hz_ measurements at four out of five time points. The single inconsistent prediction was likely caused by minor random fluctuations in experimental measurements.

Subsequent verification was performed using experimental data from Coating System No. 7. Weight coefficients obtained from its training dataset (seventh row in Table 4) were used for multi-parameter fusion evaluation with three integrated indicators: adhesion strength *A*_s_ (*λ* = 0.72), mid-frequency phase angle *θ*_10 Hz_ (*λ* = 0.65), and open-circuit potential OCP (*λ* = 0.82). Detailed prediction outputs are given in Table 6. Model-predicted grades exhibited high consistency with benchmark grades defined by|*Z*|_0.01 Hz_. Comparative evaluation using the simplified two-indicator combination (*θ*_10 Hz_ and *A*_s_) also yielded consistent results. This confirmed that the model supported flexible parameter selection and reliable coating-degradation evaluation without requiring the full set of input indicators.

Additional validations on Coating System No.5 and No.6 are summarized in Table 7 and Table 8, respectively. The model predictions generally matched the benchmark grades derived from |*Z*|_0.01 Hz_ measurements, demonstrating stable predictive performance across these extra coating systems. Table 9 summarizes the dataset statistics for all ten coating systems, listing parallel specimens and measurement points per coating system. The associated table note further provides the total number of measurement samples and their distribution across different performance grades.

Building on these individual-case validations, systematic cross-verification was conducted across all ten coating systems listed in Table 1. The overall mean fused hold-out accuracy of the proposed model reached approximately 80%, with seven out of ten systems achieving accuracy ≥ 80%. Relatively lower accuracy for individual systems mainly arose from limited sample quantity and raw-data fluctuations. For instance, System No. 8 delivered the lowest accuracy (≈73%) owing to sparse and noisy measurements. It is noteworthy that final evaluation accuracy depends heavily on the quality and stability of raw experimental datasets.

## 6. Conclusions

A multi-view deep-learning integrated framework was proposed to construct a comprehensive quantitative evaluation model for organic coating protective performance, based on abundant accelerated corrosion experimental data from various coating systems. This study established a feasible research route and a complete evaluation algorithm suite, providing an effective technical reference for multi-parameter quantitative assessment of coating degradation under laboratory-simulated marine corrosion conditions.

Low-frequency impedance modulus (|*Z*|_0.01 Hz_) was selected as the unified benchmark indicator for five-tier grading of coating performance. Open-circuit potential, mid-frequency phase angle, and coating adhesion served as optional auxiliary parameters, adaptable to test conditions and evaluation needs. An improved Pearson correlation algorithm analyzed intrinsic correlations between degradation degree and performance parameters, quantitatively deriving weight coefficients for electrochemical and mechanical indices. A multi-view deep neural network embedded with a weighted-voting fusion mechanism was then constructed to achieve accurate multi-parameter comprehensive grading with satisfactory accuracy across tested coating systems.

The proposed model overcomes the limitations of traditional single-indicator methods and enables multi-dimensional quantitative characterization of coating barrier deterioration. Experimental validation based on laboratory-accelerated test datasets confirmed reliable prediction accuracy and promising cross-system generalization potential within the studied experimental scenarios. Notably, overall evaluation precision heavily relies on data quality; dataset scale and integrity largely determine prediction reliability, and sufficient high-quality samples are essential for stable and credible model outputs.

Future work will expand and optimize this framework by incorporating new evaluation parameters such as quantitative visual indicators of blistering, corrosion degree, glossiness, and color difference, combined with low-frequency impedance data to form a coupled benchmark. Coating water absorption and porosity will be added as auxiliary indexes. Following the same core methodology, future studies will quantify correlations and weights of new parameters and adopt the multi-view weighted-voting fusion strategy for more comprehensive and precise assessments. Continued model optimization will further enhance prediction accuracy and improve its potential practical engineering value.

## Figures and Tables

**Table 1:** The metal/coating systems and their test environments.

No.	Substrate (Pretreatment)	Coating System	Test Environment
1	Steel (Sa 2.5)	Solvent-free epoxy	3.5% NaCl solution (RT)
2	Steel (Sa 2.5)	Solvent-free epoxy	3.5% NaCl solution (40 °C/12 h + 25 °C/12 h)
3	Steel (Sa 2.5)	Waterborne epoxy	3.5% NaCl solution (RT)
4	Steel (St3)	Waterborne epoxy	3.5% NaCl solution (RT)
5	Steel (Sa 2.5)	Modified epoxy	3.5% NaCl solution (RT)
6	Steel (Sa 2.5)	Modified epoxy	Neutral salt spray (NSS)
7	Steel (Sa 2.5)	Modified epoxy	3.5% NaCl solution (40 °C/12 h + 25 °C/12 h)
8	Steel (Sa 2.5)	Phenolic epoxy	3.5% NaCl solution (40 °C/12 h + 25 °C/12 h)
9	Steel (Sa 2.5)	Phenolic epoxy	Neutral salt spray (NSS)
10	Al alloy (St3)	Waterborne epoxy	3.5% NaCl solution (RT)

**Table 2:** Quantitative correlation between |*Z*|_0.01 Hz_ and coating protective performance.

|*Z*|_0.01 Hz_/Ω·cm^2^	Coating Protective Performance Grade
>1 × 10^9^	Excellent
1 × 10^8^−1 × 10^9^	Good
1 × 10^7^−1 × 10^8^	Fair
1 × 10^6^−1 × 10^7^	Poor
<1 × 10^6^	Failure

**Table 3:** Grading threshold intervals of characteristic parameters corresponding to five performance tiers for Coating System 1.

Characteristic Parameter	Grading Interval				
	Excellent	Good	Fair	Poor	Failure
*θ*_10 Hz_ /°	>50	40 ~ 50	20 ~ 40	10 ~ 20	<10
OCP/V_SCE_	>−0.1	−0.13 ~ −0.1	−0.15 ~ −0.13	−0.4 ~ −0.15	<−0.4
*A*_s_/MPa	>8	7 ~ 8	6 ~ 7	5 ~ 6	<5

**Table 4:** Weighting factors (*λ*) results of characteristic performance indicators for all coating systems.

Coating System No.	*A*_s_	*θ*_10 Hz_	OCP
1	0.93	0.60	0.37
2	0.79	0.63	0.41
3	0.89	0.65	0.33
4	0.81	0.59	0.33
5	0.76	0.60	0.74
6	0.85	0.58	0.71
7	0.72	0.65	0.82
8	0.78	0.66	0.57
9	0.88	0.64	0.88
10	0.65	0.72	0.92

**Table 5:** Model prediction results based on *A*_s_, *θ*_10 Hz_ and OCP data of Coating System No.1.

Testing Time/d	2	9	27	89	124
|*Z*|_0.01 Hz_ /Ω∙cm^2^	2.2 × 10^10^	1.8 × 10^9^	2.6 × 10^8^	1.2 × 10^7^	5.5 × 10^5^
Benchmark grade from |*Z*|_0.01 Hz_	I	I	II	III	V
Prediction by *A*_s_, *θ*_10 Hz_ and OCP					
Probability distribution of multi-level predictions	I: 0.533	I: 0.437	I: 0.256	I: 0.199	I: 0.163
	II: 0.201	II: 0.294	II: 0.432	II: 0.186	II: 0.162
	III: 0.260	III:0.260	III: 0.300	III: 0.493	III: 0.422
	IV: 0.002	IV: 0.003	IV: 0.006	IV: 0.086	IV: 0.148
	V: 0.004	V: 0.005	V: 0.006	V: 0.036	V: 0.104
Prediction by model	I	I	II	III	III
Prediction by *A*_s_ and *θ*_10 Hz_					
Multi-grade prediction probability distribution	I: 0.554	I: 0.446	I: 0.258	I: 0.209	I: 0.188
	II: 0.190	II: 0.297	II: 0.453	II: 0.177	II: 0.186
	III: 0.249	III:0.247	III: 0.277	III: 0.499	III: 0.484
	IV: 0.002	IV: 0.003	IV: 0.005	IV: 0.077	IV: 0.092
	V: 0.005	V: 0.006	V: 0.007	V: 0.038	V: 0.051
Model predicted grade	I	I	II	III	III

Note: Grade I: Excellent, II: Good, III: Fair, IV: Poor, V: Failure.

**Table 6:** Model prediction results based on *A*_s_, *θ*_10 Hz_ and OCP of Coating System No.7.

Testing Time/d	1	77	136	178	240
|*Z*|_0.01 Hz_ /Ω∙cm^2^	1.2 × 10^10^	1.9 × 10^8^	1.1 × 10^8^	1.0 × 10^7^	4.3 × 10^6^
Benchmark grade from |*Z*|_0.01 Hz_	I	I	II	III	IV
Prediction by *A*_s_, *θ*_10 Hz_ and OCP					
Multi-grade prediction probability distribution	I: 0.696	I: 0.523	I: 0.212	I: 0.203	I: 0.197
	II: 0.244	II: 0.401	II: 0.459	II: 0.210	II: 0.164
	III: 0.054	III: 0.064	III: 0.313	III: 0.567	III: 0.616
	IV: 0.001	IV: 0.004	IV: 0.005	IV: 0.007	IV: 0.008
	V: 0.004	V: 0.009	V: 0.011	V: 0.013	V: 0.015
Prediction by model	I	I	II	III	III
Prediction by *A*_s_ and *θ*_10 Hz_					
Multi-grade prediction probability distribution	I: 0.698	I: 0.511	I: 0.319	I: 0.306	I: 0.264
	II: 0.212	II: 0.375	II: 0.557	II: 0.317	II: 0.241
	III: 0.081	III:0.095	III: 0.100	III: 0.346	III: 0.308
	IV: 0.002	IV: 0.006	IV: 0.008	IV: 0.010	IV: 0.137
	V: 0.007	V: 0.013	V: 0.017	V: 0.020	V: 0.050
Prediction by model	I	I	I	III	III

Note: Grade I: Excellent, II: Good, III: Fair, IV: Poor, V: Failure.

**Table 7:** Model prediction results based on *A*_s_, *θ*_10 Hz_ and OCP data of Coating System No.5.

Testing Time/d	1	77	136	178	244
|*Z*|_0.01 Hz_ /Ω∙cm^2^	4.2 × 10^10^	3.5 × 10^8^	1.1 × 10^8^	2.9 × 10^7^	2.8 × 10^7^
Benchmark grade from |*Z*|_0.01 Hz_	I	II	II	III	III
Probability distribution of multi-level predictions	I: 0.696	I: 0.523	I: 0.212	I: 0.203	I: 0.197
	II: 0.244	II: 0.401	II: 0.459	II: 0.210	II: 0.164
	III: 0.054	III: 0.064	III: 0.313	III: 0.567	III: 0.616
	IV: 0.001	IV: 0.004	IV: 0.005	IV: 0.007	IV: 0.008
	V: 0.004	V: 0.009	V: 0.011	V: 0.013	V: 0.015
Model predicted grade	I	I	II	III	III

Note: Grade I: Excellent, II: Good, III: Fair, IV: Poor, V: Failure.

**Table 8:** Model prediction results based on *A*_s_, *θ*_10 Hz_ and OCP data of Coating System No.6.

Testing Time/d	16	60	108	230	304
|*Z*|_0.01 Hz_ /Ω∙cm^2^	6.8 × 10^9^	1.3 × 10^9^	5.9 × 10^8^	1.7 × 10^7^	3.6 × 10^6^
Benchmark grade from |*Z*|_0.01 Hz_	I	I	II	III	IV
Probability distribution of multi-level predictions	I: 0.622	I: 0.376	I: 0.494	I: 0.178	I: 0.171
	II: 0.184	II: 0.286	II: 0.269	II: 0.162	II: 0.156
	III: 0.127	III: 0.265	III: 0.161	III: 0.534	III: 0.212
	IV: 0.054	IV: 0.057	IV: 0.060	IV: 0.095	IV: 0.428
	V: 0.012	V: 0.015	V: 0.016	V: 0.031	V: 0.032
Model predicted grade	I	I	I	III	IV

Note: Grade I: Excellent, II: Good, III: Fair, IV: Poor, V: Failure.

**Table 9:** Dataset statistics of the ten coating systems used for model training and validation.

System No.	Parallel Specimens	Measurement Points
**1**	14	44
**2**	10	30
**3**	10	49
**4**	10	32
**5**	5	71
**6**	9	85
**7**	9	71
**8**	7	35
**9**	9	48
**10**	10	50
**Total**	**93**	**515**

Note: Based on |Z|_0.01 Hz_ thresholds in {{float-placeholder-materials-19-03969-t002}}, the 515 measurement samples consist of 225 Excellent (Grade I), 108 Good (Grade II), 109 Fair (Grade III), 39 Poor (Grade IV) and 34 Failure (Grade V). Since each per-parameter sub-model is trained independently, the effective sample size differs across sub-model views: approximately 433 samples for the θ_10 Hz_ sub-model, 518 for the OCP sub-model, and 51 for the adhesion-strength (*A_s_*) sub-model. The small sample size for adhesion originates from its destructive nature and low measurement frequency.

## Data Availability

The original contributions presented in this study are included in the article. Further inquiries can be directed to the corresponding author.
